# 6, 058例肺癌患者病理类型和临床流行病学特征的分析

**DOI:** 10.3779/j.issn.1009-3419.2016.03.03

**Published:** 2016-03-20

**Authors:** 仁锋 张, 岩 张, 丰标 温, 恺 吴, 松 赵

**Affiliations:** 450052 郑州，郑州大学第一附属医院胸外一科，郑州市胸部肿瘤重点实验室 Department of Thoracic Surgery, the First Affiliated Hospital, Zhengzhou University, Zhengzhou Key Laboratory of Thoracic Oncology, Zhengzhou 450052, China

**Keywords:** 肺肿瘤, 流行病学, 病理学, Lung neoplasms, Epidemiology, Pathology

## Abstract

**背景与目的:**

肺癌的流行病学特征通常随时间、地域、人群分布的变化而发生改变，本研究拟通过回顾性分析郑州大学第一附属医院2012年-2014年住院的原发性支气管肺癌患者的病理类型特点及临床流行病学特征，以初步了解近年来肺癌的流行趋势。

**方法:**

收集2012年-2014年郑州大学第一附属医院住院的诊断为原发性支气管肺癌并登记为河南地区常住人口的病例，对患者的性别、年龄、城乡来源、吸烟史、饮酒史、地区、手术情况和病理类型等临床资料进行比对分析。

**结果:**

收集肺癌病例共6, 058例，其中2012年1, 495例，2013年2, 070例，2014年2, 493例。肺癌患者男女比例2012年-2014年分别为2.26:1、2.29:1、2.20:1（χ^2^=0.367, *P*=0.832）.发病年龄以60岁-69岁年龄段居多，占35.72%，男女患者各年龄段病理类型差异具有统计学意义（χ^2^=109.848, *P*＜0.001）。2012年-2014年不同病理类型肺癌患者的构成比差异有统计学意义（χ^2^=25.344, *P*=0.013）。男女性肺癌患者均以腺癌为主，在各类病理类型中分别占37.64%和73.63%，差异具有统计学意义（χ^2^=562.382, *P*＜0.001）。各年龄段肺癌患者中腺癌所占比例分别为60.62%、56.59%、49.84%、45.15%、47.03%和41.25%，差异具有统计学意义（χ^2^=48.886, *P*＜0.001）。豫北地区腺癌的构成比最高，占55.95%。在城市与农村患者中，各病理类型构成比差异具有统计学意义（χ^2^=29.732, *P*＜0.001）。吸烟患者中以鳞癌居多，占38.39%。饮酒患者中也以鳞癌居多，占37.37%。接受手术治疗的患者占15.40%。

**结论:**

近年来腺癌在所有类型肺癌中所占比例呈上升趋势，鳞癌呈下降趋势。男性肺癌患者以腺癌最常见。腺癌在中青年患者高发，鳞癌在男性患者中高发并与吸烟饮酒关系密切。

## Abstract (en)

**Background and objective:**

The epidemiology of lung cancer changes with time, region, and population. The pathological and clinical characteristics of patients with primary bronchial lung cancer were retrospectively analyzed to understand the epidemic trend of lung cancer in recent years.

**Methods:**

We reviewed the clinical data of patients with primary bronchogenic carcinoma. These patients, who are permanent residents of Henan province, were treated in the First Affiliated Hospital of Zhengzhou University from 2012 to 2014. The distributions of gender, age, urban/rural residency, smoking history, drinking history, operation history, and histological types of tumor were compared among the patients and analyzed.

**Results:**

A total of 6, 058 cases of lung cancer were collected, including 1, 495 cases in 2012, 2, 070 cases in 2013 and 2, 493 cases in 2014. The proportions of male to female patients with lung cancer were 2.26:1, 2.29:1 and 2.20:1 in 2012 to 2014, respectively (χ^2^=0.367, *P*=0.832). The age of onset was 60-69 years old, which accounted for 35.72% of the patients, and exhibited statistically significant differences between male and female patients (χ^2^=109.848, *P* < 0.001). Moreover, differences in the pathological types of patients with lung cancer were statistically significant in 2012-2014 (χ^2^=25.344, *P*=0.013). Lung adenocarcinoma accounted for 38.5% and 73.63% in male and female patients, respectively, and the difference was statistically significant (χ^2^=562.382, *P* < 0.001). Adenocarcinoma also accounted for 60.62%, 56.59%, 49.84%, 45.15%, 47.03% and 41.25% in all ages of patients with lung cancer; the difference was statistically significant (χ^2^=48.886, *P* < 0.001). Furthermore, the proportions of various pathological types in rural and urban patients were statistically significant among the five regions of Henan Province. The proportions of squamous cell carcinoma were the highest in smoking and drinking patients, accounting for 38.39% and 37.37%, respectively. About 15.4% of the patients included in the study received surgical treatment.

**Conclusions:**

The proportion of adenocarcinoma in all types of lung cancer has increased in recent years, whereas the incidence of squamous cell carcinoma has declined. Adenocarcinoma is common in male patients with lung cancer as well as in young-and middle-aged patients. Squamous cell carcinoma is associated with smoking and drinking in male patients.

肺癌是严重危害人类生命健康的恶性肿瘤之一^[1]^，也成为当今世界范围内最常见的恶性肿瘤^[2]^。据统计2012年约新增肺癌患者180万并造成159万人死亡，其中中国约占统计肺癌病例的1/3以上^[3]^。肺癌的死亡率和发病率在世界各个地区都增加，但流行病学特征随时间、地域、人群分布的变化而发生改变^[4]^。本研究通过收集2012年-2014年郑州大学第一附属医院住院的诊断为原发性支气管肺癌并登记为河南地区常住人口的病例，对患者的性别，年龄，城乡来源，吸烟史，饮酒史，地区，手术情况和病理类型等临床资料进行比对分析，旨在初步了解近年来河南地区原发性肺癌的病理类型的变化特征及临床流行病学特点。

## 1 资料和方法

### 1.1 资料

来自郑州大学第一附属医院2012年-2014年登记住院病案，均为原发性支气管肺癌并登记为河南地区常住人口。同一患者多次入院以首次诊断作为统计时间并按一次住院处理，均通过胸水、痰液、肺穿刺、纤维支气管镜及手术等方法获得病理学依据，同时排除无病理学依据的病例（已于当地医院明确病理诊断转入本院患者也列入统计范围之内）。病理类型依照2004年版世界卫生组织（World Health Organization, WHO）肺部肿瘤组织学分类标准分型，本文共分为7类：腺癌（adenomatous, AD）、鳞状细胞癌（squamous cell carcinoma, SCC）、小细胞癌（small cell carcinoma, SCLC)、肉瘤样癌（sarcomatoid carcinoma, SC）、大细胞癌（large cell carcinoma, LCC)、腺鳞癌（adenosquamous carcinoma, ASC）和其他类型癌（else），混合癌按照其主要成分进行统计。收集内容包括患者性别、年龄、地区、吸烟史、饮酒史、城乡来源、手术情况和病理类型。

### 1.2 统计学方法

使用Statistic 6.0统计软件分析数据，组间比较采用χ^2^检验或者*Fisher*确切概率法，*P*＜0.05为差异有统计学意义。

## 2 结果

### 2.1 病例、性别的分布及动态变化

2012年-2014年肺癌患病人数逐年增多，其中男性4, 192例，女性1, 866例，男女之间比例为2.24:1。不同年份肺癌患者男女构成比之间进行比较，行卡方检验差异无统计意义（χ^2^=0.367, *P*=0.832）。显示2012年-2014年间肺癌患者男女之间分布无明显变化（表 1）。

**1:** 6, 058例肺癌患者病例分布情况和性别分布 Characteristics and gender distribution of 6, 058 patients with lung cancer

Year	Total	Male	Female	Gender-ratio
2012	1, 495	1, 036	459	2.26:1
2013	2, 070	1, 441	629	2.29:1
2014	2, 493	1, 715	778	2.20:1
Total	6, 058	4, 192	1, 866	2.24:1

### 2.2 年龄和性别分布

6, 058例原发性支气管肺癌中，不同年龄段肺癌患者所占比例行*Pearson*卡方检验，差异具有统计学意义（χ^2^=109.848, *P*＜0.001）。其中60岁-69岁患者占35.72%，该年龄段男女患者分别占各组病例数的37.21%（1, 560例）及32.37%（604例）。并且随着年龄段梯度的增加，男女性别比也不断增大，＜40岁年龄段男女性别比最小为1.08:1，≥80岁年龄段男女比例最大为4.33:1（表 2）。

**2:** 6, 058例肺癌患者年龄与性别分布 Age and gender distribution of 6, 058 patients with lung cancer

Age (year)	Total	Male	Female	Gender-ratio
≤39	193	100	93	1.08:1
40-49	857	499	358	1.39:1
50-59	1, 591	1, 089	502	2.17:1
60-69	2, 164	1, 560	604	2.58:1
70-79	1, 093	814	279	2.92:1
≥80	160	130	30	4.33:1
Total	6, 058	4, 192	1, 866	2.24:1

### 2.3 不同年份与病理类型的分布

在2012年-2014年间，6, 058例不同病理类型肺癌患者的构成比进行比较，行*Pearson*卡方检验，有统计学差异（χ^2^=25.344, *P*=0.013）。其中，腺癌在2012年-2014年的构成比分别是44.41%、48.88%和51.18%，差异有统计学意义（χ^2^=17.170, *P*＜0.001）；鳞癌的构成比分别为28.36%、25.07%和24.07%，差异具有统计学意义（χ^2^=9.339, *P*=0.009）；小细胞癌的构成比分别是21.67%、20.48%和19.82%，呈下降趋势，差异无统计学意义（χ^2^=1.978, *P*=0.372）；肉瘤样癌、大细胞癌、腺鳞癌和其他癌的构成比均无统计学差异（表 3）。

**3:** 6, 058例肺癌患者病理类型的分布 The distribution of pathological types in 6, 058 cases of patients with lung cancer

Item	AD	SCC	SCLC	LSC	LCC	ASC	Else
Year							
2012	664 (44.41%)	424 (28.36%)	324 (21.67%)	7 (0.47%)	2 (0.13%)	6 (0.40%)	68 (4.55%)
2013	1, 012 (48.89%)	519 (25.07%)	424 (20.48%)	5 (0.24%)	5 (0.24%)	15 (0.72%)	90 (4.35%)
2014	1, 276 (51.18%)	600 (24.07%)	494 (19.82%)	13 (0.52%)	5 (0.20%)	8 (0.32%)	97 (3.89%)
*χ*^2^	17.17	9.339	1.978	2.305	-	4.119	1.151
*P*	0.001	0.009	0.372	0.316	0.877^*^	0.128	0.562
Gender							
Male	1, 578 (37.64%)	1, 439 (34.33%)	945 (22.54%)	23 (0.55%)	11 (0.26%)	19 (0.45%)	177 (4.22%)
Female	1, 374 (73.63%)	104 (5.57%)	297 (15.92%)	2 (0.11%)	1 (0.05%)	10 (0.54%)	78 (4.18%)
*χ*^2^	669.446	562.382	34.788	6.124	-	0.185	0.006
*P*	0.001	0.001	0.001	0.013	0.120^*^	0.667	0.94
Age (year)							
≤39	117 (60.62%)	13 (6.74%)	41 (21.24%)	1 (0.52%)	0 (0.00%)	2 (1.04%)	19 (9.84%)
40-49	485 (56.59%)	120 (14.00%)	206 (24.04%)	1 (0.12%)	2 (0.23%)	6 (0.70%)	37 (4.32%)
50-59	793 (49.84%)	358 (22.50%)	367 (23.07%)	12 (0.75%)	3 (0.19%)	4 (0.25%)	54 (3.39%)
60-69	977 (45.15%)	634 (29.30%)	440 (20.33%)	6 (0.28%)	5 (0.23%)	10 (0.45%)	92 (4.25%)
70-79	514 (47.03%)	357 (32.66%)	165 (15.10%)	4 (0.37%)	1 (0.09%)	7 (0.64%)	45 (4.12%)
≥80	66 (41.25%)	61 (38.13%)	23 (14.38%)	1 (0.63%)	1 (0.63%)	0 (0.00%)	8 (5.00%)
*χ*^2^	48.886	162.423	36.382	-	-	-	18.128
*P*	< 0.001	< 0.001	< 0.001	< 0.135^*^	< 0.653^*^	< 0.315^*^	0.003
Region							
Eastern Henan	923 (45.99%)	538 (26.81%)	439 (21.87%)	5 (0.25%)	3 (0.15%)	8 (0.40%)	91 (4.53%)
Western Henan	270 (47.12%)	125 (21.82%)	141 (24.61%)	4 (0.70%)	2 (0.35%)	4 (0.70%)	27 (4.71%)
Southern Henan	442 (45.71%)	268 (17.37%)	202 (20.89%)	6 (0.62%)	0 (0.00%)	5 (0.52%)	44 (4.55%)
Northern Henan	362 (55.95%)	124 (19.17%)	127 (19.63%)	3 (0.46%)	2 (0.31%)	2 (0.31%)	27 (4.17%)
Central Henan	955 (51.23%)	488 (26.18%)	333 (17.86%)	7 (0.38%)	5 (0.27%)	10 (0.54%)	66 (3.54%)
*χ*^2^	28.342	22.529	16.587	3.562	-	-	3.231
*P*	< 0.001	< 0.001	0.002	0.467	< 0.305^*^	< 0.832^*^	0.520
^*^The number is too few, so we use *Fisher's* exact test, not *χ*^2^ value. AD: adenomatous; SCC: squamous cell carcinoma; SCLC: small cell carcinoma; SC: sarcomatoid carcinoma; LCC: large cell carcinoma; ASC: adenosquamous carcinoma							

### 2.4 不同性别与病理类型分布

男女性肺癌患者均以腺癌为主，在各类病理类型中分别占37.64%和73.63%，差异具有统计学意义（χ^2^=562.382, *P*＜0.001）（表 3），显示女性肺癌患者以腺癌为高发类型。鳞癌患者的男女构成比为93.26% *vs* 6.74%，显示鳞癌在男性患者中发病明显。小细胞癌以男性为主，男女构成比为76.09% *vs* 23.91%，但女性患者中小细胞癌在各类肺癌中占15.92%，高于鳞癌的5.57%。男性患者中小细胞癌占22.54%，低于鳞癌的34.33%（图 1）。

**1:**
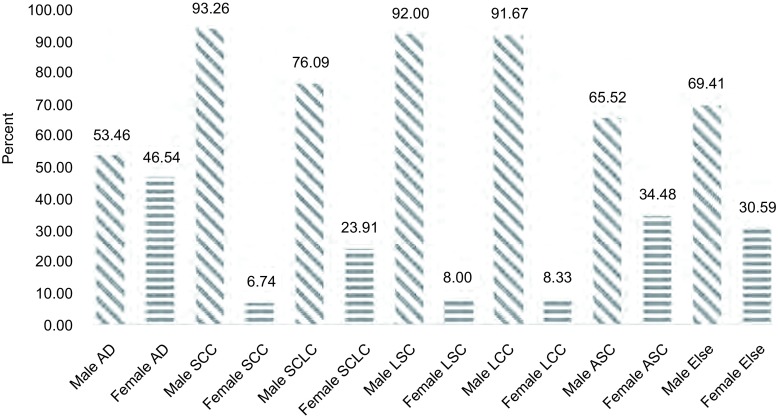
肺癌患者不同性别的病理类型分布（%） Distribution of pathology in different gender (%)

### 2.5 不同年龄段与病理类型的分布

不同年龄段的肺癌患者中，60岁-69岁患者占35.72%，比例最高。≥80岁患者占2.64%，比例最低。各年龄段肺癌患者中腺癌所占比例分别为60.62%、56.59%、49.84%、45.15%、47.03%和41.25%，行卡方检验，差异具有统计学意义（χ^2^=48.886, *P*＜0.001）。各年龄段肺癌患者中鳞癌构成比行卡方检验，差异具有统计学意义（χ^2^=162.423, *P*＜0.001）。各年龄段肺癌患者中小细胞癌构成比行卡方检验，差异具有统计学意义（χ^2^=36.382, *P*＜0.001）（表 3）。

### 2.6 不同地区与病理类型分布

各地区中腺癌占各病理类型患者比例分别为45.99%、47.12%、45.71%、55.95%和51.23%，行卡方检验，差异具有统计学意义（χ^2^=28.342, *P*＜0.001）。各地区中鳞癌占各病理类型患者比例分别为26.81%、21.82%、27.71%、19.17%和26.18%，行卡方检验，差异具有统计学意义（χ^2^=22.529, *P*＜0.001）（表 3）。

### 2.7 城乡与病理类型分布

在城市与农村患者中，各病理类型构成比行卡方检验，差异具有统计学意义（χ^2^=29.732, *P*＜0.001）。城市患者中腺癌占各病理肺癌的58.50%，高于农村的47.84%，行卡方检验，差异具有统计学意义（χ^2^= 21.090, *P*＜0.001）。农村患者中鳞癌占各病理类型肺癌的25.63%，高于城市的23.72%，行卡方检验，差异无统计学意义（χ^2^=0.896, *P*=0.344）。农村患者中小细胞癌占各病理类型肺癌的21.24%，高于城市的12.45%，行卡方检验，差异有统计学意义（χ^2^=21.959, *P*＜0.001）（表 4）。

**4:** 肺癌病理类型与吸烟、饮酒、手术情况、城乡之间的关系 The relationship between pathological types of lung cancer and smoking, drinking, surgery, area

Item	AD	SCC	SCLC	LSC	LCC	ASC	Else
Smoking							
Yes	663 (32.68%)	779 (38.39%)	473 (23.31%)	11 (0.54%)	5 (0.25%)	9 (0.44%)	89 (4.39%)
No	2, 288 (56.81%)	764 (18.96%)	769 (19.09%)	14 (0.35%)	7 (0.17%)	20 (0.50%)	166 (4.12%)
Drinking							
Yes	292 (33.99%)	321 (37.37%)	207 (24.10%)	5 (0.58%)	0 (0.00%)	2 (0.23%)	32 (3.73%)
No	2, 660 (51.16%)	1, 222 (37.37%)	1, 035 (19.91%)	20 (0.38%)	12 (0.23%)	27 (0.52%)	223 (4.29%)
Surgery							
Yes	516 (55.31%)	248 (26.58%)	106 (11.36%)	7 (0.75%)	1 (0.11%)	9 (0.96%)	46 (4.93%)
No	2, 436 (47.53%)	1, 295 (25.27%)	1, 136 (22.17%)	18 (0.35%)	11 (0.21%)	20 (0.39%)	209 (4.08%)
Area							
County	2, 658 (47.84%)	1, 423 (25.63%)	1, 179 (21.24%)	24 (0.43%)	11 (0.20%)	26 (0.47%)	233 (4.20%)
City	296 (58.50%)	120 (23.72%)	63 (12.45%)	1 (0.20%)	1 (0.20%)	3 (0.59%)	22 (4.35%)

### 2.8 吸烟、饮酒与病理类型分布

在吸烟和非吸烟肺癌患者中，各种病理类型构成比行卡方检验，差异有统计学意义（χ^2^= 375.020, *P*＜0.001）。吸烟患者中以鳞癌居多，占38.39%。在饮酒与非饮酒患者中，各病理类型构成比行卡方检验, 差异有统计学意义（χ^2^=111.053, *P*＜0.001）。饮酒患者中以鳞癌居多，占37.37%（表 4）。

### 2.9 手术与病理类型的分布

接受手术治疗的患者占15.40%，其中腺癌患者的手术比例为17.48%，鳞癌患者的手术比例为16.07%，SCLC的手术比例为8.53%，肉瘤样癌的手术比例为28%。各种病理类型患者中接受手术和没有接受手术治疗的构成比差异具有统计学意义（*P*＜0.001）（表 4）。

## 3 讨论

世界卫生组织国际癌症研究机构（International Agency for Research on Cancer, IARC）于2015年发布的GLOBOCAN癌症报告指出：全球2012年共新增癌症病例1, 400万并包括死亡人数820万。而中国地区新增癌症患者307万并导致约220万人死亡，占全世界总统计量的21.9%和26.8%^[3]^。我国的肺癌发病率和死亡率已居各类恶性肿瘤的第一位^[5]^。刘国华^[6]^对河南省1995年-1999年疾病监测地域人群的肺癌死亡特征进行了初步的流行病学研究，其中5年期间人群肺癌死亡潜在工作损失年数率呈逐年升高趋势。明显的地理分布差异是河南省肺癌流行病学特征之一，而肺癌已经是河南省上升最为明显的恶性肿瘤，一些城市肺癌的死亡率已经居恶性肿瘤的第一位^[7]^。而关于河南省近年来肺癌患者病理类型的分析和相关流行病学资料相对匮乏。本组资料仅从本院的患者资料分析，存在选择偏倚，但通过我们的研究希望从一个侧面反映河南以及中国中部地区肺癌发病情况。

近年来，肺腺癌的发病率速度具有明显上升的趋势^[1]^。本组资料中腺癌在2012年-2014年的构成比分别是44.41%、48.88%和51.18%，呈逐渐上升的趋势，而鳞癌的构成比呈逐渐下降的趋势，这与我国相关流行病学特征一致^[8]^。1977年Vincent等^[9]^在1, 682例肺癌患者中发现腺癌已经超过鳞癌。本组资料腺癌患者男性比例占53.46%，高于女性的46.54%，显示近年来腺癌患者男性比例已经超过女性，而这与国内某些地区报道^[8]^不一致。鳞癌患者中男性比例占93.26%，高于国内其他研究^[10]^。女性患者仍以腺癌为主，所占比例达73.63%，可能与室内烹调油烟的空气污染有关，特别是菜籽油^[11]^、二手烟和生物燃料的污染^[12]^。腺癌在≤39岁年龄段所占比重最大，随着年龄段的增大比重呈下降趋势。鳞癌在≥80岁年龄段所占比重最大，随着年龄段的增大比重呈上升趋势，可能因为年轻患者中吸烟者较少和烟龄较短，所以鳞癌发生率相对较低^[13]^。本次统计显示农村患者远远高于城市患者，可能与农村空气污染、农业生产中农药化肥接触、食品污染、冬季室内燃煤污染、房屋通风结构问题等关系密切^[14]^，也和农村进城务工人员多从事建筑、装修、矿产等职业可能有关。另一面与农民经济水平改善，健康意识提高，农村医疗保障制度健全以及本院在本地区影响力不断增大等可能有关。本组资料吸烟和饮酒均与鳞癌有密切联系，相关研究表明吸烟与肺癌的发生呈现一定的剂量-效应关系^[15]^。在河南各地区中豫北地区腺癌比例最高，豫南地区鳞癌比例最高，而国内尚没有记载该地区相关流行病学资料，本组结果可以为后续该地区其他方面流行病学调查提供基本依据。肺癌患者中接受手术治疗的患者仅占15.40%，手术治疗依然是肺癌治疗的主要手段，因为根治性手术目前仍是唯一有可能使肺癌患者获得有效治愈的治疗方式^[16]^，其中全胸腔镜肺癌手术兴起于20世纪90年代初，在Ⅰ期肺癌手术中的应用亦已得到普遍认可，并被写入肺癌的临床诊治指南^[17]^。但是很多患者明确诊断已经在中晚期，延缓了最佳治疗时间。总之加强教育宣传戒烟戒酒，在肺癌高发地区重点预防筛查，重视环境污染治理，以及重视现代肺癌诊疗技术的应用和农村地区常规体检的推行都将有利于防治肺癌。
